# Beyond consumption: a qualitative investigation of hospital clinician attitudes to receiving feedback on antimicrobial prescribing quality

**DOI:** 10.1017/ash.2022.20

**Published:** 2022-04-11

**Authors:** Gerry Hughes, Eilis O’ Toole, Una Coleman, Alida Fe Talento, Keith Doyle, Aisling O’ Leary, Colm Bergin

**Affiliations:** 1 Department of Infectious Diseases, St. James’s Hospital, Dublin, Ireland; 2 Trinity College, Dublin, Ireland; 3 Wellcome/Health Research Board Ireland Clinical Research Facility, St. James’s Hospital, Dublin, Ireland; 4 Clinical Microbiology Children’s Health Ireland (Temple Street), Dublin, Ireland; 5 Royal College of Surgeons, Dublin, Ireland; 6 Information Management Services, St. James’s Hospital, Dublin, Ireland; 7 National Centre for Pharmacoeconomics St. James’s Hospital, Dublin, Ireland; 8 School of Pharmacy Royal College of Surgeons, Dublin, Ireland

## Abstract

**Background:**

Feedback on optimal antimicrobial prescribing to clinicians is an important strategy to ensure antimicrobial stewardship (AMS) in the hospital setting.

**Objective:**

To explore the perceptions of antimicrobial prescribing feedback among clinicians in acute care.

**Study design:**

Prospective qualitative design.

**Setting:**

A large inner-city tertiary referral center in Dublin, Ireland.

**Participants:**

Clinicians were recruited from the hospital clinician population.

**Methods:**

A qualitative study was conducted with a purposive sample of multidisciplinary clinicians. Focus groups and semistructured interviews were used to collect data that were analyzed inductively to identify themes.

**Results:**

In total, 30 clinicians from medical, surgical, nursing and pharmacy professions participated in the study. We identified 5 themes: (1) antimicrobial consumption perceived as a proxy measure for prescribing quality; (2) lack of connection between antimicrobial prescribing and patient outcomes; (3) relevance and impact of antimicrobial prescribing feedback associated with professional role; (4) attitudes regarding feedback as an AMS strategy; and (5) knowledge regarding AMS, including antimicrobial prescribing quality measures.

**Conclusions:**

Focused feedback on antimicrobial prescribing, with clear goals for improvement, could serve as a useful AMS strategy among clinicians in the acute-care setting. The need for further education and training in AMS was also identified.

*“… if no one stops me, I’ll make the mistake again.”* ^1^

The principle of feedback involves describing deviations from best practice and reporting them to key stakeholders, including the originator, thereby reducing the likelihood of recurrences. However, the impact of feedback can be unpredictable and influenced by the context in which it is provided.^2,3^ Feedback is not a new concept in healthcare, and it is a key component of acute care antimicrobial stewardship (AMS) programs,^4–6^ which aim to educate and inform prescribers on optimal use of antimicrobials and highlight any aberrations from best practice. Prospective audit and feedback is a well-recognized component of AMS.^4^ In practice, this activity is frequently undertaken by infection specialists during AMS ward rounds.

Despite evidence of positive impact as part of AMS, feedback can often be poorly implemented. In their Cochrane review, Davey et al^6^ investigated the impact of feedback on antimicrobial prescribing in hospital environments. They found that although feedback was an effective enabler of prudent antimicrobial prescribing, it was described in only a minority of interventions.^6^

Peer approval has also been suggested as an important social determinant of AMS interventions, including feedback.^7^ Meeker et al^8^ and Hallsworth et al^9^ demonstrated this in their primary care studies. Another way of considering the social concept of peer comparison is that, in general, “No one wants to be a low performer.”^10^

The rational and prudent use of antimicrobials is a complex process with multiple actors^11,12^ that occurs in a multitude of different clinical environments, contexts, and settings. Designing, developing, and implementing feedback as a behavioral change and sustainment strategy is also invariably complex. Accounting for the perceptions of the target population as well as the local clinical and cultural environment is necessary to maximize the adoption and durability of feedback.^13^ Practically, this means designing feedback interventions that are meaningful to end users which in turn will increase the likelihood of their taking ownership of AMS.

In this study, we sought to identify stakeholder perceptions of antimicrobial prescribing feedback in an acute-care setting.

## Methods

### Design, setting, and participants

We conducted a prospective, qualitative study at St. James’s Hospial (SJH) a large, public inner-city tertiary-care referral center in Dublin, Ireland. The SJH AMS program was established in 2001 through a partnership between the departments of Infectious Disease, Clinical Microbiology, and pharmacy. It was not formally funded but was supported by the appointment of a single pharmacist via the Strategy for the Control of Antimicrobial Resistance in Ireland.^14^ Prospective audit and feedback was initiated on ward areas; educational programs were developed; and clinical audits were undertaken to monitor prescribing patterns. In 2015, the SJH AMS program underwent strategic, operational, and governance restructuring to reflect best practice in undertaking AMS in acute-care settings.^15^ Although the AMS team previously reported to the hospital pharmacy and therapeutics committee, it switched to hospital board reporting through the quality, safety, and improvement division. A new multidisciplinary strategic and oversight committee was formed with stakeholders from all relevant professions (including non–infection-related specialities) and executive management across the hospital.

The study was conducted between June 2019 and May 2020. Electronic and paper posters were distributed throughout the hospital to advertise the study. Clinicians across medical, surgical, nursing, and pharmacy professions were purposively recruited from the hospital clinician population to gain broad insight from key stakeholders relevant to the research aim. High-volume prescribers were not specifically recruited. Informed consent was obtained from each participant.

### Data collection

Focus groups and semistructured interviews were used to collect data and were hosted by a trained facilitator. A literature search informed the interview schedule, which was refined through consensus with the research team. It was subsequently amended iteratively, where deemed necessary, after each focus group or interview.^16^ Examples of unbiased discussion questions and follow-up questions are outlined in the Supplementary Materials (interview schedule).

To minimize the potential for power differentials among clinicians, each focus group consisted of homogenous professional groups.^17^ Participants were given the option to review their own transcriptions. A pilot focus group with 5 residents was conducted, and results were included in the final data set. No incentives were offered for participation.

The study was approved by the SJH Institutional Review Board and the SJH Research Ethics Committee.

### Template feedback instrument

**Fig. 1.**
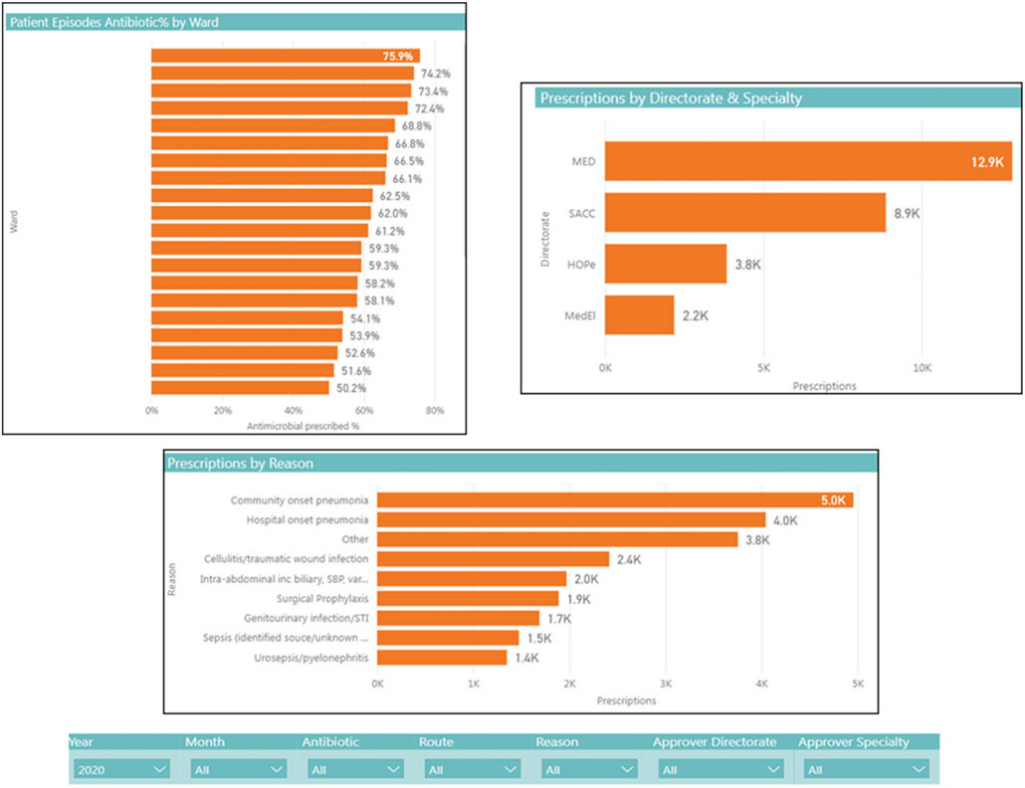
Template feedback instrument on antimicrobial prescribing (Footnotes: Figure is for illustrative purposes only and does not represent antimicrobial consumption at our institution; ward names have been redacted; directorate codes MED = medicine, SACC = surgery, anesthesiology and critical care, HOPe = hematology, oncology and palliative care, MedEl = medicine for the elderly; other acronyms: SBP = spontaneous bacterial peritonitis, STI = sexually transmitted infection)

Electronic healthcare documentation and prescribing was implemented at SJH in October 2018.^18^ A computer-generated template feedback dashboard, fed by electronic prescribing data, was developed with an in-house information technology specialist. This feedback template (Fig. 1) was presented to study participants in the context of it potentially becoming a component of the hospital’s quality and safety performance indicators.

### Data management and analysis

Focus groups and interviews were audio-recorded and transcribed verbatim. Reflective notes that contributed to data analysis were also recorded by the primary investigator (G.H). Transcriptions were completed and coded by 1 investigator (G.H.) and were reviewed by 2 additional investigators (A.O.L. and C.B.) for consistency of coded data. Data were analyzed inductively, through thematic analysis,^19^ to construct themes.

## Results

The study included 30 participants. The demographic data of the participants are listed in Table 1. Five focus groups were held with homogenous groups of hospital clinicians (n = 26), and 4 additional semistructured interviews were conducted with attending surgeons. Each focus group lasted between 23 and 49 minutes, and each interview lasted between 15 and 20 minutes.

**Table 1.** Participant Demographics

Clinician	Total	Male	Female
Resident^a^	5	4	1
Clinical nurse manager	6	0	6
Staff nurse	5	0	5
Clinical nurse specialist	2	0	2
Clinical pharmacist	2	0	2
Attending (physician)	6	3	3
Attending (surgeon)	4	4	0
**Total**	30	11	19

a Pilot-test participants.

Results are presented under descriptive theme headings punctuated by illustrative quotations from participants. We derived 5 main themes from the data (Table 2).

**Table 2.** Themes Constructed From the Data

Theme	Illustrative Quotation
Antimicrobial consumption perceived as a proxy measure for antimicrobial prescribing quality	“I suppose the things that we would be broadly interested in is obviously volume of prescriptions.”—Attending surgeon “It means, if there is a buy-in of less IV antibiotics, everyone and their mother will look it (feedback) all up and do everything and hound this doc and that doc. If it means less IVs and preparing IVs and giving IVs…”—Clinical nurse manager
Lack of connection between antimicrobial prescribing and patient outcomes	“Just what’s relevant to us… I want MY report…”—Attending physician “And from a stewardship point of view, different wards that are more inclined to use…antibiotics that we wouldn’t necessarily associate with being first line so…”—Medical resident “Why did we spend that much? Oh, because there were 7 patients on the ward and were incredibly sick. I think it (feedback) would have to be externally provided but internally checked.”—Attending surgeon
Relevance and impact of antimicrobial prescribing feedback associated with professional role	“It (feedback) should be available for everybody that wants to access it. Well, I think! Why would it not be?” Clinical nurse specialist. “… It will change your daily practice once you’ve kinda like, once, em, you can kind of reflect on it.”—Medical resident “We can keep hounding attendings and sure, we’re at nothing.”—Clinical nurse manager “I think sometimes, as well, when you don’t have senior decision makers on the ward rounds… There are no decisions made. And may not be made for 5 days!”—Clinical nurse manager “… The (medication administration) timings, and then that’s a nursing perspective that we can govern.”—Clinical nurse manager “… A resistant organism would be a reason for prescribing something that you wouldn’t normally prescribe for that indication.”—Medical resident “But looking at the number of people in the hospital who have … an antibiotic allergy, and then looking up what they’re on ….”—Attending physician
Attitudes toward feedback as an AMS strategy	“Oh, I think they (attendings) would be very positive. I mean from our perspective, we don’t handle very well somebody telling us we are not very good at operations. But we’re very good when somebody is saying you could do this (antibiotic prescribing) better.”—Attending surgeon “So when you’re prescribing too many quinolones or whatever, you need to stop doing that so it’s a slap on the wrist type of stuff is it?”—Attending physician. “You know because there are so many new things coming into the hospital, there are so many things you have to follow up on, so many things you have to do.”—Clinical nurse manager. “It’s about interpreting the data. I think if we were looking at it by ourselves what would very quickly happen is that people would go, oh that’s very interesting but I’m not sure if it’s actually relevant. We’d need context.”—Attending surgeon “So, once it’s presented at grand rounds, or like whatever, all the findings and then it kind of seeps into your mentality rather than on a daily basis on the round…that’s important.”—Medical resident “I’d use it in their (resident) training, I suppose along with the allergy and you could have other specific topics that … the residents being kept up to date on.”—Attending physician “… We’ll have a captive audience in our monthly morbidity and mortality meeting where ward staff, interns, residents, all of us sit together, look at our numbers and discuss in that…”—Attending physician
Knowledge regarding AMS, including antimicrobial prescribing quality measures	“Like, for me waiting list times and times to scan, I’m monitoring that myself cos it’s relevant to me. I’m not going to go looking for antibiotic data.”—Attending physician “Length of stay and, em, discharge lounge use … transfers out of ICU…”—Clinical nurse manager “We need to understand these KPIs (key performance indicators) better. Like you know, compliance with duration of agent with local policy. I don’t know what it is.”—Clinical nurse manager “Am, so I think people might not be aware that their prescribing, or their team’s prescribing is out of keeping.”—Medical resident “Because I think that what you’d find … that it (feedback) was only being accessed by those who were interested in antimicrobial prescribing.”—Medical resident “I know without looking anything up, what specialty will have the wrong antibiotic for a very prolonged period of time, you know? So it’s just recurrent. But it is very much people’s user preference. It’s hard to change that.”—Clinical nurse specialist “And I mean people who are in subspecialty areas … would I’m sure prescribe off label a lot more. So (feedback) is probably more relevant to those individuals.”—Attending physician

### Theme 1: Antimicrobial consumption perceived as a proxy measure for antimicrobial prescribing quality

Many participants associated antimicrobial prescribing quality with consumption and other issues such as the burden of intravenous administration or individual prescriber preferences. We observed little or no reference to measures of antimicrobial prescribing quality such as guideline conformance or use of restricted agents.

### Theme 2: Lack of connection between antimicrobial prescribing and patient outcomes

Some participants were mostly interested in their own data. Conversely, surgeons saw value in comparing antimicrobial-prescribing performance metrics because they had already provided morbidity and mortality data to international registries. Some participants questioned how their actions could make a difference if other prescribers did not change practice. However, there was no discussion of connection between antimicrobial prescribing and patient outcomes.

### Theme 3: Relevance and impact of antimicrobial prescribing feedback associated with professional role

Responses among individual participants and among professional groups varied regarding the utility of feedback on antimicrobial prescribing practice. Nurses in particular expressed frustration in attempting to affect antimicrobial prescribing change. We also noted varying opinions on what should be included in feedback, and these differed among professional groups.

### Theme 4: Attitudes toward feedback as an AMS strategy

We observed broad recognition that feedback would be appropriate as part of an audit strategy and that most stakeholders would be open to receiving this feedback. However, attitudes were mixed regarding antibiotic prescribing feedback as an AMS strategy. Some participants felt that context would be required to explain prescribing feedback to some clinicians on how to improve practice. Participants also felt that feedback should be integrated into existing organizational structures in the hospital rather than introducing new communication pathways. Other participants were cognizant of ‘metric fatigue’, were dubious about publication of data relating to prescribing performance and were suspicious toward audit and feedback.

### Theme 5: Knowledge regarding AMS, including antimicrobial prescribing quality measures

Although participants were aware of quality metrics in healthcare, there was less awareness of antimicrobial prescribing quality measures. Furthermore, antimicrobial prescribing practice was generally not a priority for their department. Also, certain professional groups were identified in terms of prescribing habits.

## Discussion

Our findings highlight the importance of engaging with key AMS stakeholders in acute care. Such a strategy is important in the development and implementation of feedback as a complex healthcare intervention.^20,21^ It was clear from the themes identified in this study that most stakeholders were not aware of AMS quality indicators. Participants mostly referred to antimicrobial consumption as the marker of prescribing quality. The most efficient method for feedback delivery identified by participants was through their own individual learning and development pathways. Additional findings indicated a need for further education and training in AMS.

Although education and training are not the only solutions to rational antimicrobial use, they are essential components of any acute-care AMS program.^4^ Such education and training should reinforce the concept of performance indicators as standardized measures of healthcare quality that shifts the focus from consumption.

Recent research has highlighted the importance of the nursing profession in acute-care AMS.^22,23^ As the largest professional workforce in hospitals, nurses have greater contact time with patients, more than other healthcare professionals. As such, they are ideally placed as AMS change agents. In this study, however, nurses harbored reservations about influencing antimicrobial prescribing habits. From a professional perspective, they expressed concern regarding how their role would extend to advising prescribers to optimize antimicrobial therapy and how prescribers would perceive nurses who would do this. These nuances are similar to those found by Broom et al^24^ in their qualitative study of clinicians’ perceptions of acute-care AMS in an Australian hospital.

Although some participants were open to the prospect of receiving feedback on their antimicrobial prescribing, others were suspicious that it would appear punitive. This finding highlights a culture where feedback can be negatively perceived. Preservation of “good manners” and “medical collegiality” is considered important in the context of noninterference with professional autonomy, which may conflict with optimization of antimicrobial prescribing through feedback.^25^ This point is important for the design of feedback interventions and reinforces the need for close stakeholder engagement.

We also observed mixed reactions from participants on comparing feedback metrics between prescribers or services, despite peer comparison previously proving successful as an AMS strategy.^9,26^

Antimicrobial prescribing is regarded as a highly autonomous act by prescribers.^25^ Some participants did acknowledge that, if identified as ‘outliers,’ they would work toward bringing themselves back in line with good practice. However, most were not prepared to have this prescribing critically appraised in an open manner. Self-monitoring was suggested as a better approach with the need for tangible goals to assist with modifying prescribing practice.

Recognizing the importance of careful intervention implementation, participants were asked how best to deliver feedback. Rather than create a new information dissemination pathway, participants felt that feedback should be integrated into existing multidisciplinary and other meetings to maximize its exposure and to engage stakeholders. Taking advantage of existing meetings ensures efficient feedback delivery in the context of busy working environments, which participants also indicated.

Recent calls for providing antimicrobial prescribing data to clinicians has emphasized the it be done in a way that encourages self-regulating rather than ‘policing’ by AMS programs.^27,28^ Indeed, one participant in this study supported so-called AMS ‘champions’ to be nominated within services and departments to complement institutional AMS programs.

The findings of this study have been presented to the SJH AMS operational working group and to the hospital electronic healthcare and quality improvement departments. Based on discussion of the findings with these groups, an electronic data collection tool to capture antimicrobial prescribing data during AMS ward rounds is currently being designed for integration into patient health records. An adapted feedback instrument (based on Fig. 1), incorporating these data, will be targeted toward antimicrobial prescribing stakeholders. It will provide feedback on the quality of prescribing within services as benchmarked against Irish antimicrobial prescribing indicators.^29^

### Strengths and limitations

Qualitative research provides a method of enquiry that reaches beyond the potential limitations of quantitative investigation to provide a deeper understanding of problems. As such, this study will inform AMS audit and feedback operations at our institution. Triangulation of data was possible such that themes identified could be compared across the different clinician groups. This study was carried out with a small sample at a large urban public hospital in Ireland. The findings may not be generalizable to other clinical settings, such as those with AMS programs at different stages of development or those with clinician specialties not represented in our study.

### Recommendations for future research

Further studies to evaluate the utility and impact of feedback should commence through a series of quality improvement cycles with a small number of stakeholder groups. Using this method will ensure that the feedback mechanism is durable and fit for the purpose of AMS

In Ireland, almost one-third of the facilities in the acute-care hospital network are private healthcare institutions.^30^ Further research on antimicrobial prescribing feedback should be conducted in private acute-care settings and compared to findings in public hospitals.

In conclusion, the findings of this study highlight the idiosyncrasies of a specific clinical context, which must be considered to facilitate engagement of key stakeholders in AMS. Albeit the sample size was small, this work has highlighted that focused feedback data with clear goals for improvement could serve as a useful AMS strategy among clinicians in the acute care setting. Integrating prescribing feedback into the fabric of existing structures of the hospital environment is essential to ensure impact and sustainability of AMS.

## Supplementary material

For supplementary material accompanying this paper visit https://doi.org/10.1017/ash.2022.20. click here to view supplementary material
