# Supplementary material for: Beyond consumption: a qualitative investigation of hospital clinician attitudes to receiving feedback on antimicrobial prescribing quality
Source: Antimicrob Steward Healthc Epidemiol. 2022 Apr 11;2(1):e63. doi: 10.1017/ash.2022.20 (PMC9726545; doi:10.1017/ash.2022.20)
Supplement: Supplementary file 1 [file S2732494X22000201sup001.docx]

Supplementary materials: Interview schedule

Welcome, introduction and general housekeeping.

Members introduce themselves.

IMS representative gives a brief description of the new capabilities of the Cerner platform in conjunction with the BI system to visually display data on antimicrobial prescribing.

**Ask if anyone has any initial observations or thoughts to share.**

**Question 1**

What kind of information would interest you in relation to the nature of antimicrobial prescribing that occurs within your service?

- Probe: Mention the national HSE metrics as the standards to be achieved (handout)

**Question 2**

What do you think about comparisons between your prescribing data to others in a similar speciality being made available to the hospital?

- Probe: Comparisons within services (e.g. between teams in the respiratory service) or between wards

**Question 3**

How would you prefer to be notified or interact with this content?

- Probe: Would you like notification emails?
- Probe: Would you prefer to interact with the information in your own time?
- Probe: Would you prefer to view the data in real time or scheduled summary reports?

**Question 4**

Would you consider this data useful for discussion among your team meetings such as journal clubs or morbidity/mortality meetings?

- Probe: How could discussion of this data be integrated into these meetings?

Participants will then be invited to discuss any further comments they may have
